# Supplementary material for: A UMLS-based spell checker for natural language processing in vaccine safety
Source: BMC Med Inform Decis Mak. 2007 Feb 12;7:3. doi: 10.1186/1472-6947-7-3 (PMC1805499; doi:10.1186/1472-6947-7-3)
Supplement: Additional file 13 — RAP application directory. Sets up directory for RAP (RDF application for PHP) source code [file 1472-6947-7-3-S13.gz › rap/doc/phpdoc/model/ModelFactory.html]

xml version="1.0" encoding="iso-8859-1"?


Docs For Class ModelFactory


## Class ModelFactory

Description

Description |
Methods (details)

ModelFactory is a static class which provides methods for creating different types of RAP models. RAP models have to be created trough a ModelFactory instead of creating them directly with the 'new' operator because of RAP's dynamic code inclusion mechanism.

History: <LI>10-05-2004 : First version of this class.</LI>

- access: public
- author: Daniel Westphal <at d-westphal.de mail at d-westphal.de>
- version: V0.9.1

Located in /model/ModelFactory.php (line 27)

Method Summary

Description |
Methods (details)

void
&getDatasetMem
([string $graphSetId = null], string $uri)

object DbModel
&getDbModel
(object DbStore $dbStore, [string $baseURI = null])

object DbStore
&getDbStore
([string $dbDriver = ADODB\_DB\_DRIVER], [string $host = ADODB\_DB\_HOST], [string $dbName = ADODB\_DB\_NAME], [string $user = ADODB\_DB\_USER], [string $password = ADODB\_DB\_PASSWORD])

object DbModel
&getDefaultDbModel
([string $baseURI = null])

object MemModel
&getDefaultModel
([string $baseURI = null])

object MemModel
&getInfModelB
([string $baseURI = null])

object MemModel
&getInfModelF
([string $baseURI = null])

object MemModel
&getMemModel
([string $baseURI = null])

object MemModel
&getOntModel
(constant $modelType, mixed $vocabulary, [string $baseURI = null])

object MemModel
&getResModel
(constant $modelType, [string $baseURI = null])

Methods

Description |
Methods (details)

getDatasetMem (line 50)

Returns a NamedGraphSetMem.

You can supply a GraphSet name.

- access: public

void

&getDatasetMem
([string $graphSetId = null], string $uri)

- string
  $graphSetId
- string
  $uri

getDbModel (line 98)

Returns a new DbModel using the database connection supplied by $dbStore.

You can supply a base URI. If a model with the given base URI exists in the DbStore, it'll be opened. If not, a new model will be created.

- access: public

object DbModel

&getDbModel
(object DbStore $dbStore, [string $baseURI = null])

- object DbStore
  $dbStore
- string
  $baseURI

getDbStore (line 118)

Returns a database connection with the given parameters.

Paramters, which are not defined are taken from the constants.php

- access: public

object DbStore

&getDbStore
([string $dbDriver = ADODB\_DB\_DRIVER], [string $host = ADODB\_DB\_HOST], [string $dbName = ADODB\_DB\_NAME], [string $user = ADODB\_DB\_USER], [string $password = ADODB\_DB\_PASSWORD])

- string
  $dbDriver
- string
  $host
- string
  $dbName
- string
  $user
- string
  $password

getDefaultDbModel (line 80)

Returns a DbModel with the database connection defined in constants.php.

You can supply a base URI. If a model with the given base URI exists in the DbStore, it'll be opened. If not, a new model will be created.

- access: public

object DbModel

&getDefaultDbModel
([string $baseURI = null])

- string
  $baseURI

getDefaultModel (line 37)

Returns a MemModel.

You can supply a base URI

- access: public

object MemModel

&getDefaultModel
([string $baseURI = null])

- string
  $baseURI

getInfModelB (line 150)

Returns a InfModelB.

(MemModel with backward chaining inference engine) Configurations can be done in constants.php You can supply a base URI

- access: public

object MemModel

&getInfModelB
([string $baseURI = null])

- string
  $baseURI

getInfModelF (line 134)

Returns a InfModelF.

(MemModel with forward chaining inference engine) Configurations can be done in constants.php You can supply a base URI

- access: public

object MemModel

&getInfModelF
([string $baseURI = null])

- string
  $baseURI

getMemModel (line 64)

Returns a MemModel.

You can supply a base URI

- access: public

object MemModel

&getMemModel
([string $baseURI = null])

- string
  $baseURI

getOntModel (line 206)

Returns an OntModel.

$modelType has to be one of the following constants: MEMMODEL, DBMODEL, INFMODELF, INFMODELB to create a OntModel with a new model from defined type. $vocabulary defines the ontology language. Currently only RDFS\_VOCABULARY is supported. You can supply a model base URI.

- access: public

object MemModel

&getOntModel
(constant $modelType, mixed $vocabulary, [string $baseURI = null])

- constant
  $modelType
- string
  $baseURI

getResModel (line 168)

Returns a ResModel.

$modelType has to be one of the following constants: MEMMODEL,DBMODEL,INFMODELF,INFMODELB to create a resmodel with a new model from defined type. You can supply a base URI

- access: public

object MemModel

&getResModel
(constant $modelType, [string $baseURI = null])

- constant
  $modelType
- string
  $baseURI

Documentation generated on Thu, 7 Jul 2005 13:42:06 +0200 by phpDocumentor 1.3.0RC3
